# Two sexes, one genome: the evolutionary dynamics of intralocus sexual conflict

**DOI:** 10.1002/ece3.540

**Published:** 2013-05-01

**Authors:** Tanya M Pennell, Edward H Morrow

**Affiliations:** Evolution, Behaviour and Environment Group, School of Life Sciences, University of SussexFalmer, East Sussex, BN1 9QG, UK

**Keywords:** Conflict resolution, sex-specific gene regulation, sexual antagonism, sexual dimorphism, sexually antagonistic coevolution

## Abstract

As the evolutionary interests of males and females are frequently divergent, a trait value that is optimal for the fitness of one sex is often not optimal for the other. A shared genome also means that the same genes may underlie the same trait in both sexes. This can give rise to a form of sexual antagonism, known as intralocus sexual conflict (IASC). Here, a tug-of-war over allelic expression can occur, preventing the sexes from reaching optimal trait values, thereby causing sex-specific reductions in fitness. For some traits, it appears that IASC can be resolved via sex-specific regulation of genes that subsequently permits sexual dimorphism; however, it seems that whole-genome resolution may be impossible, due to the genetic architecture of certain traits, and possibly due to the changing dynamics of selection. In this review, we explore the evolutionary mechanisms of, and barriers to, IASC resolution. We also address the broader consequences of this evolutionary feud, the possible interactions between intra- and interlocus sexual conflict (IRSC: a form of sexual antagonism involving different loci in each sex), and draw attention to issues that arise from using proxies as measurements of conflict. In particular, it is clear that the sex-specific fitness consequences of sexual dimorphism require characterization before making assumptions concerning how this relates to IASC. Although empirical data have shown consistent evidence of the fitness effects of IASC, it is essential that we identify the alleles mediating these effects in order to show IASC in its true sense, which is a “conflict over shared genes.”

## Introduction

The evolutionary interests of males and females are often worlds apart. This is thought to be a result of gamete dimorphism, causing the sexes to occupy distinct reproductive roles and experience contrasting selection pressures (Trivers 1972; Parker 1979). In an ideal scenario, the sexes will adapt accordingly by expressing different trait values; however, independent evolution is constrained by shared molecular “machinery” controlling trait expression in both sexes (i.e., alleles have similar additive effects in each sex). This creates high intersexual genetic correlations (r_mf_), which may make it impossible for the sexes to reach their own trait fitness optima (Lande 1980). In such instances, an evolutionary tug-of-war over allelic expression may proceed. This is a phenomenon known as intralocus sexual conflict (IASC).

Outstanding questions concern the conditions that maintain IASC and the mechanisms capable of resolving it. A key question is whether evidence of ongoing conflict is indicative of conflict that cannot be resolved, or simply a transient evolutionary stage before resolution. The literature provides some convincing evidence that genetic and behavioral innovations can allow the sexes to independently reach optimal trait values (see section “Resolving the Conflict”). It is for this reason that dimorphic gene expression and sexual phenotype dimorphism are thought to have evolved (Lande 1980). In contrast, genetic barriers and stochastic selection pressures (see sections “You Can't Always Get What You Want” and “The Dynamics of Conflict Resolution”) may ensure that the sexes remain constrained by intersexual genetic correlations, thereby preventing resolution. Consequently, the potential for resolution (or its impediment) is likely to be population- and/or trait specific, and knowledge of why (or why not) conflicts are resolved is critical to understanding their evolutionary dynamics.

In this review, we also explore the links that exist between intra- and interlocus sexual conflict (IRSC: sexual conflict that occurs over the outcome of male–female interactions rather than shared traits; Rice and Holland 1997), as they appear to be closely associated through reciprocal interactions (summarized in Box 1). Historically, IASC was overshadowed, as attention was drawn to IRSC and the coevolutionary arms races that follow, potentially driving speciation and diversification (Chapman et al. 2003; Arnqvist and Rowe 2005; Tregenza et al. 2006); however, interactions between these two forms of sexual antagonism could have important evolutionary consequences, which have not been reviewed before. There are several possibilities, including the potential for IRSC to alter selection on traits that are shared between the sexes, thereby fueling IASC. On the other hand, IASC may prevent a trait from evolving in one sex, which could stall arms races that result from IRSC. Resolved conflict could also present an opportunity for a trait to become exaggerated in one sex, potentially causing IRSC if a novel and harmful interaction between the sexes is formed (see Box 1).

**Box 1. Interactions between intra- and interlocus sexual conflict**

**Figure 1:**
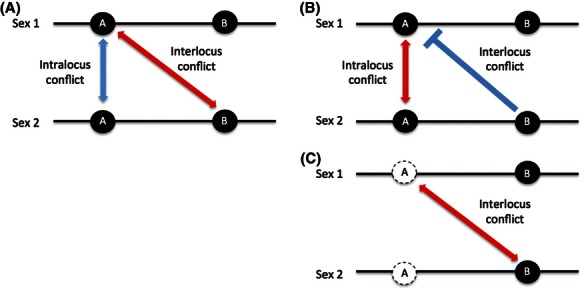
The possible interactions between intra- and interlocus sexual conflict. Loci are represented by letters (A/B) surrounded by circles (closed = existing conflict, open = resolved conflict). Selectional forces and responses to selection are represented by red and blue arrows, respectively. (A) IRSC selects on a shared trait to cause IASC. (B) IASC can prevent a trait from coevolving in response to selection caused by IRSC. (C) Resolved IASC can allow a trait to coevolve in response to IRSC, thereby enabling an intersexual arms race.

The first potential interaction to consider is how interlocus sexual conflict (IRSC) may be able to ignite IASC (Fig. 1A). Consider male mating rate as an example. Often, as mating frequency increases, male fitness is expected to increase accordingly; however, females are expected to incur relatively greater costs from multiple mating compared with males (Thornhill and Alcock 2001). This includes time and energy costs, as well as increased risk of pathogen/parasite infection, predation, and injury. Therefore, by increasing male mating rate, this could consequently promote IRSC and therefore create positive selection for females to reduce the effects of male harassment. Genes involved in mating resistance, however, could be intersexually genetically correlated. This may consequently spark IASC over resistance traits. Innocenti and Morrow (2010) also suggest another possible link between inter- and intralocus sexual conflict. They identified transcripts from sex-limited tissues that are thought to be mediating IASC, such as those expressed in accessory gland and sperm-storage organs. The authors suggest a link between the two forms of sexual antagonism because these tissues are also thought to be important in mediating male–female coevolutionary arms races that stem from IRSC (Chapman et al. 2003; Pitnick et al. 2009).

Second, if IASC over this trait remains unresolved, then counteradaptations in response to IRSC may be inhibited (Fig. 1B). In the case described above, males would be permitted to evolve toward their optimal fitness value for mating frequency, while the female resistant trait (and therefore mating rate) may be trapped at a suboptimal value. This could explain why counteradaptations in some female traits are not apparent, even though they are expected to arise. This may lead to false assumptions that females benefit from high (observed) mating frequencies, when in fact they do not.

A third interaction to consider is that which stems from resolved conflict, that is, if mechanisms arise to resolve conflict (enabling males and females to evolve independently of each other) this may allow a male trait to become exaggerated to a point where it reduces female fitness due to harmful interactions (Fig. 1C). For example, many male sperm traits are under the control of duplicate genes that are expressed solely in males (Wyman et al. 2012). As mentioned previously, this may have evolved as a way to resolve IASC. These sperm-related genes, however, are often found to be rapidly evolving under positive selection (Swanson and Vacquier 2002), which is possibly due to coevolutionary arms races between the sexes that result from IRSC. The release from IASC may thus have contributed toward these arms races. Consequently, female fitness may be reduced by IRSC in a way that is comparable to the reduction in fitness caused by IASC. This also raises questions regarding whether resolving IASC ultimately achieves net fitness benefits within a population.

We aim to bring together theoretical concepts and empirical findings to better understand the evolutionary dynamics of IASC, and to highlight fruitful avenues for future research. In this review, we take a multifaceted approach by considering the maintenance, resolution, and consequences of this evolutionary feud.

## An Ongoing Conflict

IASC is receiving an increasing amount of attention from evolutionary biologists, taking the form of various studies – both at the phenotypic and genetic level. A large body of evidence for ongoing IASC comes from correlative studies in particular. This includes hemiclonal analysis, a method developed by Rice (1996) for use in *Drosophila melanogaster*, where the direct effects of genome-wide allelic variation on sex-specific fitness can be observed via the production of “hemiclones.” Here, random individuals are taken from a source population and their genomes are expressed in random genetic backgrounds, creating many individuals of the same haplotype – analogous to fertilizing a set of clonal eggs with many sperm (Abbott and Morrow 2011). This is achieved through three distinct crosses, involving so-called “clone-generator” females (possessing a compound X chromosome, where the two copies are physically fused together, and a translocation of the major autosomes), and wild-type males. The resulting heterozygous genotype suppresses recombination between parental chromosomes and controls transmission of the male-derived complement, producing individuals that are identical across more than 99.5% of the genome. By generating multiple hemiclonal lines from one population, this provides a “snapshot” of the standing genetic variation and permits further experiments to measure the fitness of a genome in relation to the sex it is expressed in. For now, these studies are confined to the *D. melanogaster* model system (Rice 1998; Chippindale et al. 2001; Gibson et al. 2002; Pischedda and Chippindale 2006; Long and Rice 2007; Bedhomme et al. 2008; Innocenti and Morrow 2010), as there is limited scope for its application in other species. This owes to the fact that many systems lack the genetic tools necessary to force the inheritance of whole haplotypes intact (Abbott and Morrow 2011). Nevertheless, several natural hemiclones do exist in the wild, which are not currently capitalized on in the field of IASC (Abbott and Morrow 2011), and the possibility of using this method in other members of the genus *Drosophila* has not to our knowledge been explored.

Further evidence of ongoing conflict comes from studies showing reduced fitness of opposite-sex offspring. Similar to hemiclonal analysis, these correlative studies illustrate how a fit male genotype can be less fit when expressed in a female – and *vice versa*. IASC has been demonstrated in this way in a laboratory study of ground crickets (*Allonemobius socius*), where higher fitness males were shown to sire high fitness sons, but low fitness daughters (Fedorka and Mousseau 2004). Later studies of wild mountain goat (*Oreamnos americanus*) and red deer populations (*Cervus elaphus*) further demonstrate that opposite-sex offspring suffer declines in fitness (Foerster et al. 2007; Mainguy et al. 2009). Pischedda and Chippindale (2006) opted for a different approach, using hemiclonal analysis to produce high and low fitness hemiclones, and then subsequently measuring the fitness of offspring from both males and females. Consistent with IASC theory, there was a negative correlation between the fitness of parents and their opposite-sex offspring.

Artificial selection regimes can also be applied to demonstrate ongoing conflict. Mokkonen et al. (2012) artificially selected on male testosterone levels in bank voles (*Myodes glareolus*), leading to increased male reproductive success, but declines in female reproductive success. Earlier work by Morrow et al. (2008) enforced gender-limited selection independently in each sex through experimental constraints on reproductive success in *D. melanogaster*. This resulted in a decline in the net adult fitness of the nonselected sex relative to the selected sex. Prasad et al. (2007) found parallel evidence in the same system*,* by imposing gender-limited selection in a different way – the X and autosomal chromosomes were experimentally forced to cosegregate as haplotypes and thus to be transmitted from father to son. This novel method prevented female-specific selection in most of the haploid genome*,* which could then be expressed in males and females, and the sex-specific fitness consequences of male-limited evolution characterized. In an attempt to uncover the behavioral phenotype behind this sexual antagonism, Bedhomme et al. (2008) observed reproductive and feeding traits in the males and females expressing these genomes. Female feeding rate and attractiveness were found to decline with expression of chromosomes subject to male-limited evolution, findings consistent with IASC. In contrast, male courtship intensity declined but their ability to secure matings remained unchanged, a more surprising result. Although there are other possible explanations, one consistent with IASC theory is that males became more efficient at securing matings, thus courtship intensity could be reduced (Bedhomme et al. 2008). This could have a positive overall effect on the lifetime fitness of males; however, experimental design may have led to unrealistic measures of male reproductive behaviors. For example, the intensity and quality of male courtship were not investigated and there was a difference in the competitive environment and generation time during which reproductive behavior and fitness were observed.

It is evident from the studies cited above that IASC is widespread among organisms with separate sexes. Yet, the genomic distribution and fitness effects of antagonistic loci remain largely unknown. Theory suggests that such an allele can exist on any chromosome (autosome or sex chromosome) when its fitness benefits to one sex outweigh the costs imposed on the opposite sex (Rice 1984; but see Fry 2010); however, for XY systems, it is predicted that there are more sexually antagonistic alleles on the X chromosome than elsewhere (Gibson et al. 2002; Lindholm and Breden 2002; Fitzpatrick 2004; Tower 2006; Innocenti and Morrow 2010; but see Fry 2010). Specifically, male-benefit recessive alleles and female-benefit dominant alleles are expected to accumulate here. If we consider X-linked recessive alleles that are male benefit, they are always expressed in males (because males are hemizygous in XY systems), but expressed in only half of all females (those that are homozygous for this allele). Consequently, there is weak selection against them in females, because the benefits are exposed to selection more frequently than the costs (Rice 1984). Similarly, female-benefit dominant alleles will also be selected to accumulate on the X chromosome, because they are expressed two thirds of the time in females, but only one third of the time in males (Rice 1984). Following Rice's theory, the patterns of expression that occur on the X chromosome could also enable a sexually antagonistic allele to be selected for, even if the costs imposed on one sex exceed the benefits to the other. Under these circumstances, they could cause net fitness loss within a population. It may therefore be expected that sexually antagonistic alleles of greatest fitness effect may be found on the X chromosome, rather than autosomes. This could explain observations by Pischedda and Chippindale (2006) and Foerster et al. (2007), who found that high fitness sires had low fitness daughters, whereas there was no correlation between sire and son fitness. We might expect such a pattern to arise if the most significant antagonistic fitness effects are caused by X-linked alleles, which consequently will not be inherited from father to son.

Rice (1984) modeled changes in the frequency of X-linked antagonistic alleles over time. Due to the fitness costs imposed on the opposite sex, such alleles never reached fixation within a population, but were instead maintained at a stable equilibrium frequency. Recently, Dean et al. (2012) characterized the dynamics of an X-linked sexually antagonistic allele empirically, which before now had only ever been predicted by theory. They artificially created a male-benefit sexually antagonistic allele that resided on the X chromosome and reduced female fitness when expressed in a homozygous state. After 23 generations, this allele increased in frequency from 3% to 8%. Additional populations were created where the initial frequency of the antagonistic allele was at a higher percentage (35–85%). After three generations, the frequency of this allele declined. This novel approach has provided a valuable insight into the maintenance of IASC, showing that the X chromosome is capable of harboring antagonistic alleles at an equilibrium frequency, much like Rice (1984) had anticipated.

A recent model by Mullon et al. (2012) also considered how genetic drift might differentially affect the maintenance of antagonistic alleles on the autosomes and sex chromosomes. For XY systems, it is often assumed that genetic drift affects the X chromosomes to a much greater extent due to their smaller effective population size (Vicoso and Charlesworth 2009). It could therefore be expected that the X chromosome might actually harbor fewer antagonistic alleles, due to selection being less efficient in the face of drift; however, Mullon et al. (2012) argue that genetic variation at antagonistic loci is actually more likely to be maintained on the X chromosomes than the autosomes; this is due to increased reproductive variance in males, which subsequently increases the effective population size of the X. The opposite is thought to be true in ZW systems, where females are the heterogametic sex. Under these circumstances, the Z chromosome will have a low effective population size compared to the autosomes because of the lower reproductive variance in females (Mullon et al. 2012). Consequently, there may be a contrast between the genomic location of antagonistic loci in XY and ZW systems, with the sex chromosomes harboring more sexually antagonistic alleles in XY systems.

A better insight into the genetic basis of IASC could be achieved through the application of molecular and genomic tools. Recent technological advancements in sequencing methods are laying the foundations for such fine-scale genomic studies (Davey et al. 2011), which will allow the location and function of sexually antagonistic genes to be identified. This would be an important development, as genetic studies of this kind are currently scarce (Williams and Carroll 2009). Recent research by Smith et al. (2011) provides some evidence for a specific gene which may be mediating IASC in *D. melanogaster*. The gene identified (autosomal gene, cypbg1) confers DDT resistance when upregulated by the insertion of a transposable element (DDT-R). Previously, females that expressed cypbg1 were found to have higher fitness, even in the absence of DDT (McCart et al. 2005). Nevertheless, before the use of DDT as an insecticide, the DDT-R allele existed in natural populations at low frequency. This raises questions concerning why the DDT-R allele did not rise to high frequency in spite of fitness benefits to females. Smith et al. (2011) suggest this may be a result of sexual antagonism, as they find some evidence (although inconsistent) for a fitness cost to males of upregulating cypbg1; however, the authors state that the ultimate cost of DDT-R in males was unclear because its effect on fitness depended on genetic background, and therefore selection against DDT-R due to sexual antagonism may not be strong enough to explain its low frequency before the introduction of DDT.

In order to identify more extensive patterns of intralocus conflict, the application of modern genomic tools may be useful in some organisms. This could facilitate the identification of correlations between genes, sex, and fitness, which could potentially provide strong evidence for the occurrence of IASC if followed up by mechanistic studies. Innocenti and Morrow (2010) made some progress toward identifying the molecular basis of sexually antagonistic genome-wide variation. They fitted a regression model to test for associations between gene expression, fitness and sex in *D. melanogaster*. Use of the FlyAtlas database (a resource developed by Chintapalli et al. 2007) also allowed the authors to identify tissue-specific patterns of sexually antagonistic transcripts. A total of 8% of *D. melanogaster* transcripts were shown to be sexually antagonistic, with enrichment in all tissues except for the gonads. The pattern described may result from the gonads' specific regulatory mechanisms and a lack of correlation between the genes expressed here and those expressed in other tissues. These results are interesting because they imply ongoing sexual antagonism through almost the entire body. Also, the proportion of transcripts shown to be sexually antagonistic relative to the proportion that was related in some way to adult fitness was large (∼60%). It is also likely to be a conservative estimate, as conflict over different traits may arise at other life stages due to dramatic changes in selection pressures throughout development.

Despite phenotypic and molecular evidence for ongoing battles between the sexes over shared traits, the evolutionary importance of these conflicts is still debated (Bonduriansky and Chenoweth 2009; Van Doorn 2009). The damaging fitness effects of IASC are clear to see, but a pertinent question remains – is this simply a precursor to conflict resolution, or an indication of unresolvable conflict? There is no direct approach for addressing this question; however, we will attempt to find some answers within the accumulating array of literature on IASC resolution.

## Resolving the Conflict

### An easy solution?

Conflict resolution is an active topic for biologists studying IASC. This owes to the uncertainty of whether traits experiencing IASC will eventually reach resolution, or whether they will remain in this state indefinitely. In order to address this question, we need to consider the possible mechanisms of resolution. This will help us to dissect traits on an individual basis to predict their evolutionary fates and the consequences for whole-organism fitness.

An abundance of theoretical work suggests that conflict could be resolved via a number of mechanisms, which together or in isolation, would relieve the gender load that arises when the sexes are displaced from their fitness optima. Sexual dimorphism is suspected to represent conflict resolution and is thought to be caused by underlying changes in the genetic architecture of a particular trait, which then permits males and females to evolve along their own independent trajectories. This occurs when underlying genetic changes cause the intersexual genetic correlation (r_mf_) to deviate from 0. In fact, a negative correlation between r_mf_ and sexual dimorphism was identified across most traits in the fly, *Prochyliza xanthostoma* (Bonduriansky and Rowe 2005a).

To test whether sexual dimorphism represents a robust resolution to IASC, Tigreros and Lewis (2011) applied artificial selection to a dimorphic trait (body size) in opposing directions to each sex. They were able to demonstrate that once dimorphism evolves, it can be irreversible under short-term selection; thus signifying a resistant resolution to sexual conflict. It may then be reasonable to assume that as the evolution of sexual dimorphism is biologically widespread (Darwin 1871), then perhaps conflict resolution is too.

This might hold true to some extent; however, sexual antagonism has in fact been found to affect even highly dimorphic traits (Pischedda and Chippindale 2006; Long and Rice 2007; and see Bedhomme et al. 2008). Furthermore, a review of selection estimates for 89 traits taken from 34 species reinforces these findings (Cox and Calsbeek 2009). As Cox and Calsbeek (2009) state, if the extent of dimorphism does not match up to the fitness peaks of the two sexes, then sexual dimorphism will not be an indication of permanent conflict resolution. More support is provided by Innocenti and Morrow (2010), who identify existing conflict over traits with sexually dimorphic gene expression. In their study, almost 92% of the genes identified were found to be sex biased in expression (Innocenti and Morrow 2010), and only 8% of these were actually shown to be sexually antagonistic. As conflict may be absent for many of these dimorphic transcripts, this could be an indication of widespread conflict resolution. To predict whether these patterns have evolved under positive selection in response to IASC between the sexes, it is necessary to assess the fitness consequences of sex-specific expression levels. Indeed, a look at genome-wide transcription profiles reveals that a considerable amount of sex-biased gene expression is related to sex-specific functions with positive fitness effects (Connallon and Clark 2011a). Therefore, we could envisage that the dimorphic gene expression patterns shown by Innocenti and Morrow (2010) might have evolved as a mechanism to resolve conflict; however, for some genes identified in this study, sex-specific transcription did not always predict sex-specific functions or fitness consequences. This highlights how some transcriptional differences between the sexes may not have evolved directly in response to IASC. These studies provide consistent evidence that although sexual dimorphism could theoretically permit resolution, its use as a signature of resolved conflict should be avoided.

Nevertheless, there are many theoretical examples of how sex-specific gene expression (which may lead to sexual dimorphism) could resolve IASC. One way to achieve this is via sex-specific hormonal cascades or modifiers (Rice 1984). For example, secondary sexual trait expression is determined by testosterone levels in vertebrates (Mougeot et al. 2004; Blas et al. 2006) and titers of juvenile hormone in insects (Emlen et al. 2006), both of which differ between the sexes. These hormone levels will subsequently affect the induction of intracellular signaling that leads to changes in gene transcription. Concentrations of regulatory proteins that target specific genes can also affect the level of gene transcription. These regulatory proteins play an important role in *D. melanogaster* and *Caenorhabditis elegans*, for example*,* by initiating sex-specific developmental pathways (Yi and Zarkower 1999; Yi et al. 2000). There are outstanding questions regarding the birth of such gene expression patterns, as dimorphism could either result from the repression or gain of gene expression in one sex relative to the other (Williams et al. 2008). Nevertheless, there are a handful of studies addressing this question, where the authors have been able to identify genes involved in regulating sexual dimorphism, and predict an ancestral state of monomorphic expression for some traits (Emlen et al. 2007; Williams et al. 2008; Moczek and Rose 2009; Williams and Carroll 2009; Khila et al. 2012). A phylogenetic analysis of wing pattern evolution in butterflies also found evidence that for some traits, sex-limited gene expression occurred simultaneously as the trait arose in a lineage; whereas, for other traits there was an ancestral state of dimorphic expression, followed by the subsequent loss of expression in one sex (Oliver and Monteiro 2011).

An additional mechanism for controlling sex-specific gene expression is through alternative splicing (McIntyre et al. 2006). Here, sex will determine the final protein form that is produced from a shared coding region in the genome. This is a posttranscriptional process, where the RNA produced from a single gene is spliced in alternate ways through the joining of different exon combinations. McIntyre et al. (2006) conducted a genome-wide analysis of alternative splicing in *D. melanogaster,* discovering that at least 12% of all genes are spliced in this sex-specific manner. Although empirical data are lacking, it is possible that the patterns of sex-biased alternative splicing described here may have evolved to resolve IASC.

The translocation of genes to sex chromosomes could also facilitate sex-limited gene expression (Charlesworth and Charlesworth 1980; Rice 1984; Bachtrog 2006). It is thought that some male-benefit, female-detriment genes have been translocated from autosomes to the Y chromosome, for example (Bachtrog 2006), consequently enabling males to evolve independently of females (in species where females are the homogametic sex). In order for this to resolve conflict, however, females must not require the translocated gene for functional purposes. As this is unlikely for most genes, a favorable alternative hypothesis is that genes are duplicated, translocated, and then undergo sex-limited gene expression (Ellegren and Parsch 2007; Baur et al. 2008; Connallon and Clark 2011b; Gallach and Betrán 2011). In this scenario, duplications initially produce additional gene copies with identical function, but they can later be released from the ancestral function by evolving freely through mutation and selection (Wyman et al. 2012). When followed by sex-limited expression, this can subsequently allow the sexes to diverge in their trait values. The duplications produced can also be translocated to nonsex-chromosome locations (Mank 2009), with sex-specific modifiers evolving to control their expression on autosomes too. A recent analysis of gene expression by Wyman et al. (2012) found that these duplicate pairs are typically male biased in expression. This is suggested to be a product of sperm competition, as this can create strong sexual selection pressures on male traits, such as those related to ejaculate function (and may also have implications for IRSC; see Box 1).

Genomic imprinting presents another possible mechanism to alleviate IASC through sexual dimorphism (Day and Bonduriansky 2004; Patten and Haig 2008). Imprinting relies on changes to DNA methylation patterns that occur during gametogenesis in parents and affect the expression of genes in offspring. The best-known examples are igf and igf2, growth factors that are known to be silenced when inherited paternally (Barlow 1995; Ferguson-Smith and Surani 2001). Simulations indicate that this pattern could arise due to IASC, as long as the benefits of imprinting an antagonistic allele in one sex outweigh the costs of doing so in the other (Day and Bonduriansky 2004). The possibility for an imprinting modifier allele to invade a population in this way is also heavily dependent on dominance, as shown in simulations by Cleve and Feldman (2007). Their study built upon a previous model by Day and Bonduriansky (2004), where only additive variation for fitness was considered. Despite these findings, for imprinting to fully resolve conflict it would be necessary for parents to imprint genes in a gamete karyotype-specific manner. For example, males should imprint genes so that male-benefit sexually antagonistic alleles are switched off in X-bearing sperm only. This would enable males to increase the fitness of sons, without detrimentally affecting the fitness of daughters. This mechanism would include imprints on autosomes that were dependent on whether they are found in X or Y sperm. Despite this theoretical requirement for resolution, the occurrence of imprinting in this manner is yet to be proven empirically. So far, 80 genes are recognized as being imprinted in mammals (Morison et al. 2005), although others propose that this figure could actually exceed 600 (Luedi et al. 2005). Imprinting therefore presents another potential mechanism with capabilities of resolving conflict on a genome-wide scale, but one that lacks empirical support.

Sexual dimorphism has also been shown to increase for some traits as a result of condition dependence, by weakening r_mf_ (Bonduriansky and Rowe 2005b). Condition dependence is expected to evolve for traits that are under strong sexual selection, which as a result become exaggerated to a point where they are costly to produce – hence the expression of these traits comes to reflect condition (Rowe and Houle 1996). If the level of condition dependence of a trait becomes unequal between the sexes, then this may permit the elaboration of a trait in only one sex, consequently exaggerating the degree of sexual dimorphism; however, Bonduriansky and Rowe (2005b) do not quantify the fitness consequences of sexual dimorphism through condition dependence; therefore, its ability to resolve conflict was not clear. It is also necessary to explore the genetic mechanisms facilitating this as it is also unclear how trait r_mf_ affects the potential for condition dependence (Bonduriansky and Rowe 2005b).

Rather than confronting the genetic basis of IASC, some species appear to have evolved an alternative way to mask the effects of sexually antagonistic genes – sex ratio adjustment (SRA). It is conceivable that this strategy presents a means of partially resolving IASC when it is not possible to achieve sex-limited gene expression via changes to trait genetic architecture. A study conducted in the wild and follow-up laboratory investigation revealed how side blotched lizards, *Uta stansburiana*, can choose sperm depending on the phenotype of their mate (Calsbeek and Sinervo 2004). This enables females to select the sex of their offspring as a remarkable way to diffuse IASC. For instance, females mated to larger males produce more sons because size is positively correlated to male fitness, but negatively correlated to female fitness. In accordance, a small sire results in increased production of daughters. Both sexes benefit from this since it presents an opportunity to maximize the fitness of their progeny in the face of antagonistic alleles. There are parallel findings in brown anoles, *Anolis sagrei* (Calsbeek and Bonneaud 2008; Cox and Calsbeek 2010); fruit flies, *D. melanogaster* (Connallon and Jakubowski 2009), and barn owls, *Tyto alba* (Roulin et al. 2010).

Katsuki et al. (2012) focused on SRA in broad-horned flour beetles. Interestingly, the sex of offspring produced by a female depended on her own fitness, rather than that of her mate. A low fitness female produced opposite-sex offspring, whereas higher fitness females increased the production of daughters. Why the fitness of their mate had no effect on offspring ratio was not clear, but by basing offspring ratio on recognition of their own fitness, females could increase their inclusive fitness and that of their mates. Although lacking any pertinent evidence, they suggest females could alter their hormone levels to determine offspring sex.

A simple model was also developed by Blackburn et al. (2010) to explore the circumstances under which SRA could evolve. Providing that sufficient genetic variation exists at SRA loci, then SRA is expected to evolve rapidly. They note that while they only looked at a single gene, SRA is equally likely to evolve in the presence of many sexually antagonistic genes if it results in a net increase in fitness.

As well as allowing us to understand the selection pressures leading to sex ratio adjustment, these studies reinforce the argument that IASC can in fact have evolutionarily important outcomes. Nevertheless, to obtain a more complete picture, the proximate mechanisms leading to SRA require much greater empirical attention.

It would seem that IASC could be eliminated through both genetic and strategic innovations; however, this is not to say that sexual antagonism for every trait may be so easily resolved. In particular, there is much to learn about the genetic mechanisms behind the evolution of sexual dimorphism and how these work to alleviate IASC (Rhen 2000; Rice and Chippindale 2001, 2002; Day and Bonduriansky 2004; Bonduriansky and Rowe 2005b); especially in the face of strong intersexual genetic correlations (Lande 1980) or when pleiotropic genes are involved (Badyaev 2002; Ellegren and Parsch 2007; Van Doorn 2009). Moreover, despite expectations that sex-biased gene expression could rapidly evolve to diminish sexual conflict (Reeve and Fairbairn 1996, 2001; Van Doorn 2009), others describe this conclusion as premature (Stewart et al. 2010). This is supported by evidence that low levels of sexual antagonism can exist for traits that appear to be sexually dimorphic (Harano et al. 2010). Perhaps, IASC in some traits can only ever be partially resolved, with a simmering level of sexual antagonism always maintaining fitness levels below optima for the sexes. To understand, this requires a look at the potential barriers to conflict resolution, for which there is some convincing evidence.

## “You Can't Always Get What You Want”

As previously mentioned, r_mf_ is negatively correlated with many sexually dimorphic traits (Bonduriansky and Rowe 2005a), owing to the fact that when the sexes share the same genetic architecture for a trait it becomes difficult for them to become sex limited in expression and thus to become sexually dimorphic. Measurements indicate that r_mf_ for many traits is high (Lande 1980; Meagher 1994; Roff 1997; Merila et al. 1998; Delph et al. 2004; Mank 2007; Chenoweth et al. 2008), which also implies that it could be difficult to resolve IASC through sexual dimorphism. While some propose that mutations with sex-biased effects could accumulate given enough time, which would weaken the r_mf_ and permit the evolution of sex-limited gene expression (Van Doorn 2009), others contend this. Stewart et al. (2010) state that the evolution of some mechanisms to achieve sex-limited gene expression (gene duplication, alternative splicing) will be very slow unless the gene is already controlled by a sex-specific DNA regulatory binding site, or if a duplicated gene can be translocated to where it can be regulated in such a way. In contrast, changes involving sex-specific gene regulation might resolve IASC in a far shorter timeframe (Ellegren and Parsch 2007).

The effectiveness of gene duplication in relieving IASC could also be lessened if it consequently disrupts existing gene networks after translocation (Force et al. 1999; Gu et al. 2004; Huminiecki and Wolfe 2004; Gallach and Betrán 2011). It could also prove to be a poor resolution, as any mutations that arise will not be exposed to selection in the nonexpressing sex. This could cause mutations to accumulate in this gene, which may consequently be deleterious when expressed in the opposite sex. In other words, the mutational load will be doubled as the gene is only exposed to selection half of the time (Morrow et al. 2008). Furthermore, after duplication and translocation, genes could be indirectly selected via covariance with other genes, causing IASC to reoccur in a trait where it was once temporarily resolved (Hosken 2011).

Pleiotropic interactions between those genes involved in sexual antagonism, and those that are not, could be a common impediment to conflict resolution (Badyaev 2002; Ellegren and Parsch 2007; Van Doorn 2009). Harano et al. (2010) suggest a role for pleiotropy in mediating IASC in broad-horned flour beetles (*Gnatocerus cornutus*). Here, resolved conflict appears to be depicted by the stark contrast between a male's exaggerated mandible size and a female's absence of this exaggeration. To explore this further, Harano et al. (2010) used artificial selection to increase male mandible size; but while there was no correlated response in female mandibles, female fitness declined simultaneously as male fitness increased. A proximate explanation for the reduction in female fitness is that a reduction in female abdomen size, which also occurred in response to selection on male mandible size, affected egg production, and lifetime reproductive success. This provides some support for the idea that there may be genetic covariance between mandible size in males and a trait that is sexually antagonistic. Despite conclusions made by Harano et al. (2010), it should also be considered that similar effects on female fitness might also result from IRSC; for example, an increase in male mandible size may have promoted a harmful interaction between the sexes that directly reduced female fitness.

The scale of pleiotropic effects is not fully resolved, but Fitzpatrick (2004) found a majority of genes to be pleiotropic in *D. melanogaster*. Genes were randomly sampled from FlyBase (http://www.flybase.net) and labeled as pleiotropic if they contributed to two or more traits. Of the genes studied here, 78% were deemed pleiotropic, and most were putatively sexually selected but not preferentially sex linked. Under the premise that this pattern reflects that found across the genome, pleiotropy could present a significant obstacle to whole-genome conflict resolution. Mank et al. (2008) provide further evidence for pleiotropy as a constraint to resolution, although using tissue specificity in expression as a proxy for actual pleiotropy, with tissue-specific genes deemed less pleiotropic than nonspecific genes. The specificity of genes was then compared to the level of sex-biased expression. A consistent relationship was identified between sex-biased gene expression and tissue specificity in both mice and chickens. This is expected to represent resolved conflict, as these genes may have been able to achieve sex-biased gene expression due to lack of pleiotropic constraint. The results also suggest that most pleiotropic genes are those experiencing sexual antagonism, which is supported by the link between pleiotropy and absence of sex-biased expression; however, the validity of this proxy is debatable since genes can be expressed in multiple tissues and serve the same function in each of them. Conversely, a gene that is expressed in only one tissue may function in completely different ways throughout development. Also, as mentioned previously, caution should be taken when using sex-biased expression as a proxy for resolved conflict.

It is clear that IASC could be more easily resolved for some traits than others, and that a gender load may always exist due to underlying genetic architecture. As discussed, there are multiple genetic obstacles that contribute toward making genome-wide resolution practically impossible, especially as many genes serve multiple functions as well as the antagonistic trait (Ellegren and Parsch 2007). There is, however, an important gap in our knowledge of the genetic basis of sexual antagonism. This could be filled through studies that focus on the genes underlying this conflict and the genetic architecture of sexually dimorphic traits that appear to represent conflict resolution. This is relevant because there is no clear evidence for how sex-specific regulation evolves for genes that are under sexually antagonistic selection (Mank 2009).

## The Dynamics of Conflict Resolution

Mank et al. (2011) took an interesting perspective on IASC, linking sex chromosome evolution to dosage compensation and sexual antagonism. Sex chromosome evolution may be a product of sexual antagonism, allowing sex-limited expression of genes to diffuse conflict; however, a consequence could be that some genes on the X chromosome are hypertranscribed in the heterogametic sex in an attempt to compensate for having only one X chromosome. This in itself sets the stage for IASC, as it can result in overexpression of genes in the homogametic sex and subsequent counteradaptations to reduce transcription levels, which could be an important factor when considering the maintenance of sexual antagonism and prevention of resolution.

Heterogeneity in sex-specific optima (Van Doorn 2009) could also weaken selection for conflict resolution, because the fitness consequences of possessing an allele would become variable over space and time in each sex. For instance, sexual conflict environment could alter the selection pressures acting on antagonistic alleles and stall conflict resolution (Brommer et al. 2012). This could occur if a female trait to minimize the cost of mating (i.e., arising from IRSC) increased fitness in environments with a high exposure to males, but caused a decrease in fitness in low exposure environments (Brommer et al. 2012). The physical environment could also affect trait optima for the sexes (Mokkonen et al. 2012), with heterogeneous conditions potentially causing parallel selection pressures to those found by Brommer et al. (2012).

Condition dependence could work in a similar way. Although Bonduriansky and Rowe (2005b) found that condition dependence could resolve conflict, they note that this may depend on the function, costs, and genetic architecture of the sexually antagonistic trait. They also showed that intersexual genetic correlations for condition dependence could evolve, which may in fact cause sexual conflict itself. From another perspective, perhaps this alters the dynamics of selection for any kind of conflict resolution. Intersexual genetic correlations for condition dependence, for example, will mean that any selection on a trait will be dependent on both male and female condition, and how gene expression and fitness is subsequently affected. Therefore, such variable selection pressures for sex-limited gene expression could maintain sexually antagonistic alleles and render conflict resolution less probable. This is comparable to the variable selection pressures caused by environmental heterogeneity. This is an interesting avenue for future research, particularly as there is no clear evidence for whether condition dependence could eliminate or exaggerate IASC (Bonduriansky and Rowe 2005b).

Condition dependence and environmental heterogeneity appear to maintain sexually antagonistic alleles within a population. As this should theoretically create selection for conflict resolution, it therefore seems paradoxical that they could also act to prevent resolution altogether; however, if a trait is condition dependent, or affected by environmental heterogeneity, then at one time IASC and selection for resolution may be strong, yet at other times IASC and selection for resolution could weaken. Such variable selection against IASC could perhaps prevent resolution from evolving at all for some traits. We now consider some other examples of where this could apply.

In an effort to discover the conditions under which sexually antagonistic alleles can be maintained, Arnqvist (2011) used simulations to explore the effects of assortative mating by fitness. In the presence of antagonistic alleles, this translates into disassortative mating by genotype. Based on the conditions that sexually antagonistic variation was polygenic (Patten et al. 2010) and fitness exhibited sex-specific dominance (Fry 2010), matings that occurred between individuals of similar fitness were shown to maintain sexually antagonistic alleles in these simulated populations (Arnqvist 2011). As assortative mating based on phenotype is almost ubiquitous in nature, and often correlates with genetic quality, it could therefore maintain IASC in many species (Arnqvist 2011).

Further theoretical work suggests that population size could also influence the maintenance of sexually antagonistic variation (Connallon and Clark 2012). The incorporation of the effects of recurrent mutation and genetic drift into population genetic models of sexual antagonism illustrates this well. One property of antagonistic selection is that it is rendered ineffective in the face of genetic drift (Connallon and Clark 2012). By accounting for the fact that smaller populations are more susceptible to the effects of genetic drift, this means that sexually antagonistic alleles are less likely to occur under these circumstances. Sexually antagonistic alleles are therefore expected to be maintained in larger populations because antagonistic selection is able to override the effects of genetic drift, thus increasing the mean heterozygosity and contribution to fitness variance of these antagonistic loci (Connallon and Clark 2012). Interestingly, an independently derived theory also predicts that IRSC will be greater in larger, higher density populations (Gavrilets 2000), a prediction with some empirical support (Martin and Hosken 2003). Given the numerous potential links between intra- and interlocus sexual conflict (Box 1), population size may play a key role in the maintenance of sexually antagonistic alleles.

If the dynamics of population size or mating habits are constantly changing, then this may act to prevent conflict resolution, much like environmental heterogeneity or condition dependence could. Thus, although these processes are able to increase the level of sexual antagonism at times, at any point when their dynamics change, selection for conflict resolution could be reduced. This could lead to perpetual sexual antagonism without resolution ever evolving. Studying sexual conflict in species that experience stochastic environmental selection pressures and changing population dynamics could help us to understand how the intensity of sexual antagonism could change in this way, and ultimately how this may hinder or promote the evolution of conflict resolution.

## The Broader Consequences

As highlighted throughout this review, IASC could have grave impacts on population-level fitness, and attempts to resolve this conflict may strongly influence gene movements and chromosomal arrangements. Less apparent, however, are the broader consequences of IASC and its widespread evolutionary significance for animal behavior and life history traits.

As mentioned, sexual antagonism can dramatically impact offspring sex ratio (Calsbeek and Sinervo 2004; Calsbeek and Bonneaud 2008; Connallon and Jakubowski 2009; Cox and Calsbeek 2010; Roulin et al. 2010; Katsuki et al. 2012). This is an important outcome in itself, but there are also broader scale implications within a population to consider; changing sex ratios can profoundly affect mating behaviors and strategies (Weir et al. 2011). For example, when the sex ratio of a population becomes female biased, male competition may be reduced and aggressive interactions between the sexes might become less frequent. In contrast, a male-biased sex ratio could increase male–male competition as females become limiting. This can consequently affect sexual selection on the sexes and significantly alter their evolutionary trajectories (and potentially the intensity of IRSC; see Box 1).

Van Doorn (2009) explains how sex linkage of genes caused by sexual antagonism could have consequences for mate choice and sexual selection. Fisher's runaway hypothesis (Fisher 1958) for the exaggeration of male traits, and sexual selection based on “good genes” (Hamilton and Zuk 1982) are used as examples. These selection processes are facilitated by patterns of sex linkage (Kirkpatrick and Hall 2004) caused by IASC; however, for traits where conflict is still ongoing, runaway selection and sexual selection based on “good genes” may not work. For example, selection based on “good genes” will be less efficient because, while it allows males to be chosen on the basis of producing fit sons, any daughters produced may be of lower fitness (Pischedda and Chippindale 2006).

IASC has also been suggested to play an important role in speciation (Rice and Chippindale 2002). This may result if the gender load created by sexual antagonism causes coevolution between sexually antagonistic and gender-limited genes. It is then plausible that sexual coevolution within a population could subsequently cause allopatric populations to diverge, leading to hybrid infertility upon secondary contact. Comparatively, the role of IRSC in speciation is more established (Rice et al. 2005), but involves similar process of perpetual sexual coevolution to that predicted for IASC.

Sexual antagonism can also have implications for modes of sex determination, leading to rapid evolutionary transitions in some species (Bull 1983; Marín and Baker 1998; Haag and Doty 2005; but see Van Doorn 2009 for an extended discussion). This encompasses both environmental sex determination (ESD), where the sex of an individual is determined by environmental cues, and genetic sex determination (GSD), where genes are exclusively responsible for determining sex. In a highly stochastic environment, ESD is likely to evolve if these fluctuations have dramatic sex-dependent fitness consequences (Charnov and Bull 1977). On the other hand, GSD may be favorable under circumstances where genetic variation has sex-dependent fitness effects (Rice 1986), or where ESD would be detrimental due to fluctuations in sex ratio that are too great (Dooren and Leimar 2003).

Abbott (2010) proposes a role for IASC in promoting shifts from hermaphroditism (one sex morph) to gonochorism (two sex morphs). This could occur if IASC leads to selection for linkage between sexually antagonistic alleles and loci for sex determination, consequently resulting in the evolution of proto sex chromosomes (Bedhomme et al. 2009). A focus on groups that make frequent transitions to and from gonochorism could be useful to study this concept further (Abbott 2010).

Theoretically, populations experiencing IASC could also be at higher risk of extinction (Kokko and Brooks 2003); however, currently there is no indication that this actually occurs in nature. Moreover, Kokko and Brooks (2003) modeled the effects of IASC with the antagonistic allele reaching high frequencies and even going to fixation. This contrasts earlier theory (Rice 1984) and recent empirical work (Dean et al. 2012) where sexually antagonistic alleles are predicted to be maintained at a stable equilibrium without reaching such high frequencies. Under these circumstances, the risk of population extinction due to IASC is likely to be lower than implied by Kokko and Brooks (2003).

The role of IASC in maintaining genetic variation is also worthy of mentioning. Why we see so much genetic variation for traits that appear to be under strong selection has been the subject of longstanding debate. In theory, this should erode any additive variation that exists at such loci, as populations approach optimal trait values. Reasons proposed for the maintenance of additive variation include the following: high mutation rates, stochastic selection pressures, trade-offs, mate compatibility, and condition dependence (reviewed in Pizzari and Birkhead 2002). Rice (1984) first suggested that sexually antagonistic selection could also maintain genetic variation at selected loci; yet this remains to be proven empirically.

An interesting, and yet so far unexplored, consequence of IASC is its ability to maintain disease alleles within a population (E. H. Morrow and T. Connallon, unpubl. data). In particular, this could apply to some early-onset diseases that are sex specific in their effect. A disease allele such as this would be beneficial to one sex, but increase disease susceptibility in the other. In this sense, it could be maintained within a population despite its negative effects on health. Further investigation into this is likely to have profound effects on the approaches taken to medical research and the design of personalized medicine in healthcare.

IASC could also explain the evolutionary conundrum of aging. One theory as to why organisms senesce is that alleles which favor reproduction early in life will be favored, even if they are detrimental for fitness in the future (Hamilton 1966; Williams 1957). This pattern of selection will arise due to the inevitability of dying later in life from external causes, such as accident, disease, and predation. IASC could affect the aging process by causing early aging and shorter life spans than are optimal for the sexes. One such scenario could be an allele that is selected in males because it increases male fitness early in life. The same allele could reduce lifespan due to pleiotropic effects. If it acts in a sexually antagonistic manner by having a detrimental on female fitness early in life, it could also reduce female lifespan to a value that is far from optimal. A study of seed beetles was able to identify intersexual genetic correlations for lifespan, while also showing that the optimal balance between reproduction and lifespan was different for the sexes (Berg and Maklakov 2012). Selection for increased lifespan was positively correlated to female fitness, but reduced male fitness. In contrast, negative selection on life span increased male fitness, but caused female fitness to decline, fitting expectations of IASC theory. The difference in optimal lifespan is explained by the high cost of mating in males, which acts to reduce fitness later in life (Berg and Maklakov 2012). By extending lifespan, this likely reduced male fertilization success through some costly proximate mechanism (i.e., reducing ejaculate investment). Antagonistic selection could thus explain genetic variation for lifespan within a population.

Interactions could also occur between intra- and interlocus sexual conflict. They are both important determinants of evolutionary pathways, but this is currently an undeveloped area of research (see Box 1). Interlocus conflict is another form of sexual antagonism, which involves conflict over separate male–female traits, involving different loci. It is possible that selection created by IRSC could cause IASC itself (Morrow and Innocenti 2012), but sexually antagonistic arms races that stem from IRSC could be halted through constraints caused by IASC. Conflict resolution also appears to be important, as this could release a trait from any evolutionary constraint and allow it to evolve in response to IRSC.

Combining studies of intra- and interlocus conflict could help us to disentangle any interactions that do exist, and may help us to answer questions about how they affect fitness and evolution. We could begin by using known examples of IRSC (perhaps where sexually antagonistic coevolutionary arms races appear to have ceased), in order to identify intersexual genetic correlations and existing IASC over the traits involved.

## Future Directions

A “top down” approach to studying IASC has provided consistent evidence of its fitness effects; but to show IASC in the true sense of the term, which is “conflict over genes that are shared by the sexes,” we need to identify specific cases of sexually antagonistic alleles within a population. To do this requires fundamental knowledge of the genetic basis of fitness variation and sexual antagonism, which is currently almost entirely lacking.

It is also necessary to consider the conditions under which we expect this conflict to be resolved, so that we can predict the outcome for conflict over traits with important fitness effects. What seems to be evident is that genome-wide resolution is likely to be impossible. By identifying the genetics of conflict on a trait-by-trait basis, we can then edge closer to understanding what is constraining resolution.

Many studies use sexual dimorphism to suggest that conflict has been resolved in the focal trait(s); however, we argue that this common preconception should be avoided, as sexual dimorphism does not always indicate resolved conflict (see section “Resolving the Conflict”). Similarly, even where conflict is absent for traits that are shared by the sexes, this does not indicate that dimorphism evolved in response to past conflict. In fact, for some traits there may be no positive fitness benefits of dimorphism at all for the sexes (Connallon and Clark 2011a), suggesting that it can arise via random drift. For future studies of IASC, the fitness consequences of sexual dimorphism therefore need to be quantified before making such assumptions.

Identification of sexually antagonistic genes from both lab and field research within the same organism will also provide useful information on how the abundance and distribution of these alleles change according to selection pressures in these environments. Price and Hosken (2007) also suggest studying natural populations with skewed sex ratios and comparing them to nonbiased populations. This is because we might expect traits that are experiencing IASC to resemble the most common sex. Here, selection may be skewed in the direction of this sex and therefore the frequency of a given sexually antagonistic allele may increase.

Sexual antagonism at adult stages has been the focus of much of the research relating to IASC. However, it is an evolutionary conflict that unfolds through developmental time – it is after all also known as an “intersexual ontogenetic conflict” (Rice and Chippindale 2001). We might predict that IASC could be greater for genes that are important during adult development, as the sexes begin to fulfill their sex roles through divergent phenotypes; however, this does not mean that conflict is expected to be absent at earlier stages, as sex-specific developmental processes also occur during cell differentiation and embryonic growth. In line with this, there is some evidence that IASC is present in preadult stages (Prasad et al. 2007); however, contrasting evidence finds conflict during adult stages only (Chippindale et al. 2001; Cox and Calsbeek 2009). It would be interesting to identify the genes involved in IASC at these different life stages to make a more complete comparison.

Other opportunities for research into sexual antagonism could lie with hermaphroditic species (Abbott 2010). They present an interesting case, as sexual antagonism could operate on a different level to species with two sex morphs (gonochorists). This is curious because, although individuals are monomorphic, they still have male and female gametes and thus the potential for sexual selection to operate on fitness components (Morgan 1994) – a phenomenon termed “intraindividual sexual antagonism” (Abbott 2010), although there is no empirical evidence for this type of conflict thus far.

Finally, an extended form of genomic conflict could exist in species where different reproductive morphs or tactics exist (J. K. Abbott et al. unpubl. data), or where there is reproductive division of labor (e.g., in eusocial species). In the latter, fitness-related genes that experience sexually antagonistic selection may be constrained further by a intralocus caste-antagonism arising from selection acting on worker fitness that may differ to that operating on either of the reproductive sexes. This is distinct to the kinds of conflict typically studied in eusocial taxa, and the additional antagonistic selective pressure arising from the division of labor found in these species could add a third direction to the two-way tug-of-war envisaged between males and females; a case of “three or more castes, one genome.”
